# Investigation of certain physical–chemical features of oil recovery by an optimized alkali–surfactant–foam (ASF) system

**DOI:** 10.1007/s00396-017-4162-1

**Published:** 2017-07-28

**Authors:** S. M. Hosseini-Nasab, P. L. J. Zitha

**Affiliations:** Department of Geoscience & Engineering, Petroleum Engineering Group, Delft University of Technology, Delft, Netherlands

**Keywords:** Micro-emulsion phase behaviour, Foam stability, In situ soap, Interfacial tension (IFT), Lamellae number, Enhanced oil recovery (EOR)

## Abstract

The objective of this study is to discover a synergistic effect between foam stability in bulk and micro-emulsion phase behaviour to design a high-performance chemical system for an optimized alkaline–surfactant–foam (ASF) flooding for enhanced oil recovery (EOR). The focus is on the interaction of ASF chemical agents with oil in the presence and absence of a naphthenic acid component and in situ soap generation under bulk conditions. To do so, the impact of alkalinity, salinity, interfacial tension (IFT) reduction and in situ soap generation was systematically studied by a comprehensive measurement of (1) micro-emulsion phase behaviour using a glass tube test method, (2) interfacial tension and (3) foam stability analysis. The presented alkali–surfactant (AS) formulation in this study lowered IFT between the oil and aqueous phases from nearly 30 to 10^−1^–10^−3^ mN/m. This allows the chemical formulation to create considerably low IFT foam flooding with a higher capillary number than conventional foam for displacing trapped oil from porous media. Bulk foam stability tests demonstrated that the stability of foam diminishes in the presence of oil with large volumes of in situ soap generation. At lower surface tensions (i.e. larger in situ soap generation), the capillary suction at the plateau border is smaller, thus uneven thinning and instabilities of the film might happen, which will cause acceleration of film drainage and lamellae rupture. This observation could also be interpreted by the rapid spreading of oil droplets that have a low surface tension over the lamella. The spreading oil, by augmenting the curvature radius of the bubbles, decreases the surface elasticity and surface viscosity. Furthermore, the results obtained for foam stability in presence of oil were interpreted in terms of phenomenological theories of entering/spreading/bridging coefficients and lamella number.

## Introduction

Gas injection for enhanced oil recovery (EOR) suffers from poor sweep efficiency. Three reasons are associated with this deficiency of gas flooding: (1) segregation and gravity override due to the lower density of gas compared to oil and/or water, (2) viscous fingering due to high mobility ratio between injected gas and oil and/or water and (3) channelling through high-permeability streaks or layers in heterogeneous and layered reservoir [14]. Foam is the most effective method to alleviate all drawbacks associated with gas flooding EOR process. Foam is a dispersion of gas phase in a liquid phase, where the thin liquid films (called lamellae) between gas bubbles are stabilized by a surfactant which adsorbs onto the gas/liquid interfaces. The flow of the gas and surfactant solution through the porous media results in in situ foam generation [19, 27]. Foam can improve the volumetric sweep efficiency by drastically lowering gas mobility and increasing apparent viscosity, thus, providing a favourable mobility ratio and contacting a larger fraction of the reservoir to mitigate the effect of heterogeneity, gas segregation and viscous instability [6, 26, 32].

Foam has shown promise as a drive fluid for improved oil recovery (IOR) and EOR, particularly for shutting-off unwanted gas production in production wells, in the field applications, carbon dioxide (CO_2_) and nitrogen (N_2_) foam flooding and steam flooding [9, 20, 34]. In reservoirs with a high variation of permeability, strong foam will form in higher permeability zones leading to the diversion of the flow from high to low permeability zones [5, 18]. Foam has also been identified as an attractive alternative to polymer in alkaline–surfactant–polymer (ASP) flooding either for low permeability reservoir formations or for reservoirs with high salinity formation water [16, 36]. Foam offers better properties than polymers for conformance control issues due to the fact that foam can divert flow from high permeable regions to low permeable regions [35]. Alkaline–surfactant–foam (ASF) flooding has been developed as a recent new technique, which uses foam as a mobility control agent instead of polymer and provides low interfacial tension (IFT) to increase the capillary number [7, 17]. Others have proposed similar processes under the name of alkali–surfactant gas (ASG) or low tension gas (LTG) flooding [37, 38]. Alkali used in the ASF EOR interacts with carboxylic acids of the crude oil, where they generate in situ surfactant (soap) which reduces interfacial tension [23]. Alkali–surfactant (AS) formulations causes ultra-low IFT reduction that has led to an increase in the capillary number in order to mobilize the residual oil which is trapped by capillary forces [10–12, 42].

During field applications, foam may encounter varying conditions such as a range of oil saturations and different salinities. Therefore, foam should be designed to be stable for varying oil saturations and salinity [15]. On the other hand, because of varying residual oil saturations in the reservoir, strong foam could create a large pressure gradient which may cause a fracture in the reservoir. In cases where foam is injected into swept zones with a low oil saturation, intermediate or low stability foam in the presence of oil may be adequate. Moreover, although foam improves volumetric sweep efficiency, its microscopic displacement efficiency is low. Therefore, an understanding of how foam behaves physico-chemically under bulk conditions in an oil recovery process is of great importance.

Thus, this paper aimed to discover a synergistic effect between micro-emulsion phase behaviour and foam stability in bulk and to design a high-performance chemical system for an optimized slug/drive formulation for ASF EOR process. The study of micro-emulsion phase behaviour and of the foaming stability of selected chemicals in the absence and in the presence of model oil, with and without naphthenic acid, was first undertaken. We focused on the high molecular weight internal olefin sulfonate (IOS) surfactant for all the experiments. The focus in foam stability screening test was to specifically address the impact of the surfactant concentration, salinity, alkalinity, oil saturation, IFT and in situ soap generation. To this end, the obtained results for foam stability in the presence of oil are discussed in terms of the classical entering/spreading coefficient, lamella number and the stability of pseudo-emulsion film. Finally, afterwards, the main conclusions of this study are drawn.

## Theoretical background

### Entering, spreading and bridging coefficients and lamella number

Several mechanisms of foam/oil interaction have been suggested in the literature. Four main parameters have emerged as predictors of foam stability in the presence of oil: spreading and entering coefficients, the lamella number and pseudo-emulsion film models [13, 22]. Foam may become unstable when an oil droplet enters the gas–water interface under favourable thermodynamic conditions, leading to the rupture of the foam lamellae. The ability of an oil droplet to enter the gas–water interface is expressed by the entry coefficient (*E*) defined as follows [25]

1 $$ E={\sigma}_{gw}+{\sigma}_{ow}-{\sigma}_{og} $$

where *σ*_*gw*_ , *σ*_*ow*_and*σ*_*og*_ are the foaming solution surface tension, the IFT between the initial foaming solution/oil, and the surface tension of the oil phase, respectively. If *E* is negative, then the oil droplet cannot enter the foam interface, the oil droplet remains in the liquid phase, and there is no detrimental effect of the oil on the foam film. If *E* > 0, then it is thermodynamically favourable for oil to enter the gas–water interface. If the entry condition is favourable, then oil might spread on the gas/water interface. Attempts to correlate the spreading behaviour of oil droplets to foam destruction by oil form the basis for most of the work performed on oil destabilization mechanisms [30]. The spreading coefficient (*S*) for an oil–foam system is given by

2 $$ S={\sigma}_{gw}-{\sigma}_{ow}-{\sigma}_{og} $$

when *S* is negative, oil does not spread and instead oil droplets form lenses at the gas–water interface. For positive spreading coefficient (*S*), oil spreads over the liquid–gas interfaces, and the resulting foam film may rupture once the oil drop enters both surfaces of the lamella [39]. Under this condition, an oil droplet can span through the film lamella by making a meta-stable bridge (i.e. *B* is positive). Bridging coefficient *B* is defined as an indication of the mechanical stability of oil bridging on foam destabilization [8]

3 $$ B={\sigma}_{gw}^2+{\sigma}_{ow}^2-{\sigma}_{og}^2 $$

When the *B* coefficient is positive then the film lamella is unstable, while negative values of *B* lead to a stable film. Table 1 gives a summary of the foam stability prediction by the negative/positive signs of the *E*, *S* and *B* coefficients.

**Table 1:** Foam stability prediction by the negative/positive signs of the *E*, *S* and *B* coefficients

Entry coefficient *E*	Spreading coefficient *S*	Bridging coefficient *B*	Foam stability condition
Negative	Negative	Not defined	Stable foam
Positive	Negative	Negative	Stable foam
Positive	Negative	Positive	Unstable foam
Positive	Positive	Negative	Moderate stable foam
Positive	Positive	Positive	Unstable foam
Negative	Positive	Negative	Stable foam
Negative	Positive	Positive	Unstable foam

Schramm and Novosad [28, 29] proposed the use of another dimensionless parameter called the lamella number (*L*) to investigate foam stability in presence of oil. This parameter describes foam stability based upon oil emulsification in the foam structure and moving oil droplets into the foam lamellae. Lamella number (*L*) is defined as a ratio of the capillary pressure at plateau borders to the pressure difference across the oil–water interface

4 $$ L=\frac{\varDelta {P}_C}{\varDelta {P}_R}=\frac{r_o}{r_p}\frac{\sigma_{gw}}{{\sigma \theta}_{ow}} $$

where *r*_*o*_ is the radius of an oil droplet and *r*_*p*_ is the radius of the plateau border. They defined three types of foam depending on the value of the lamella number (*L*): type *A* foam for *L* < 1, type *B* foam for 1 < *L* < 7 and type *C* foam for *L* > 7 [28, 29]. Table 2 presents a summary of the foam stability prediction by the lamella number theory.

**Table 2:** Foam stability prediction by the lamella number theory

Type of foam	Foam stability to oil	*E*	*S*	Lamella number (*L*)
*A*	Quite stable foam	Negative	Negative	*L* < 1
*B*	Moderately stable foam	Positive	Negative	1 < *L* < 7
*C*	Quite unstable foam	Positive	Positive	*L* > 7

## Experimental materials and methods

### Materials

Brine containing sodium chloride (NaCl, Fisher Scientific) in deionized water (pH = 6.8 ± 0.1) was used to prepare the surfactant solution. The alkaline solutions were a mixture of sodium carbonate (Na_2_CO_3_) and sodium chloride that were obtained from Fisher Scientific Company with ACS purity. Nitrogen gas with purity of 99.98% was used to generate foam. Normal hexadecane (n-C16, Sigma-Aldrich) as the model oil was used to represent the oleic phase. Hexadecane as the model oil was used with and without a naphthenic acid, which was decanoic acid (99% pure, Sigma) in this study. Decanoic acid (0.25 wt%) dissolved in *n*-hexadecane was used, which gives a total acid number (TAN) of 2.2 mg KOH/g oil determined by ASTM method D664. A commercial IOS with a high number of carbon chains prepared by the Shell Chemical Company was selected. The co-solvent was a sec-butyl alcohol (SBA, Merck, 99% pure), and a concentration of 0.5 wt% was used. In this study, IOS surfactant was used for all the experimental investigations, as this type of surfactant has been shown to have a low IFT and to be a relatively stable foam [7, 10, 11]. The synthesis steps and the chemical structures of IOS surfactant were reported by Barnes et al. [1, 2]. The surfactant solution was prepared using brine containing NaCl or a blend of NaCl and Na_2_CO_3_.

### Phase behaviour and IFT measurement

**Fig. 1:**
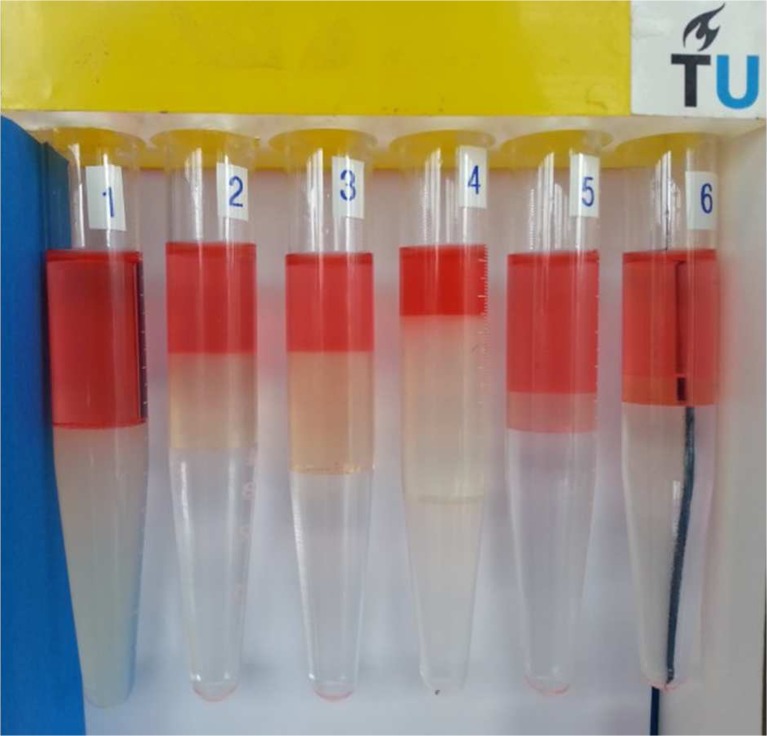
Phase behaviour of IOS and *n*-hexadecane with the glass tube salinity scan method. Salinity (NaCl) varies from left to right (1.0 to 6.0 wt%) in the surfactant solution

Samples for micro-emulsion phase behaviour studies were prepared in test tubes by adding equal amounts of aqueous surfactant solutions, co-solvent (SBA) and oleic phase (i.e. hexadecane). The thermodynamically stable micellar phase, which is clear and composed of surfactant, brine and oil, is called “micro-emulsion” [3, 24]. The samples were mixed well and were allowed to equilibrate in the atmospheric pressure. They were removed from the oven briefly several times during equilibration, shaken by hand a few times and replaced. This procedure was continued until the phase volumes remained unchanged. One example of the test tubes is shown in Fig. 1.

The phase characteristics of each system were recorded as the relative volumes of the aqueous and oleic phases and, if present, the middle phase [40, 41]. The surface tension (ST) and interfacial tension (IFT) were measured using a KSV Sigma tensiometer by the DuNouy ring method. The gas above the oil and water for ST measurement was air. The measurements were conducted for a sufficiently long time to obtain a constant value. All measurements were performed at ambient temperature (21 ± 1 °C) under atmospheric pressure. The low and ultra-low IFTs between the oil phase and water phase were measured using spinning drop tensiometer (SITE100, Kruss).

### Bulk foam stability

**Fig. 2:**
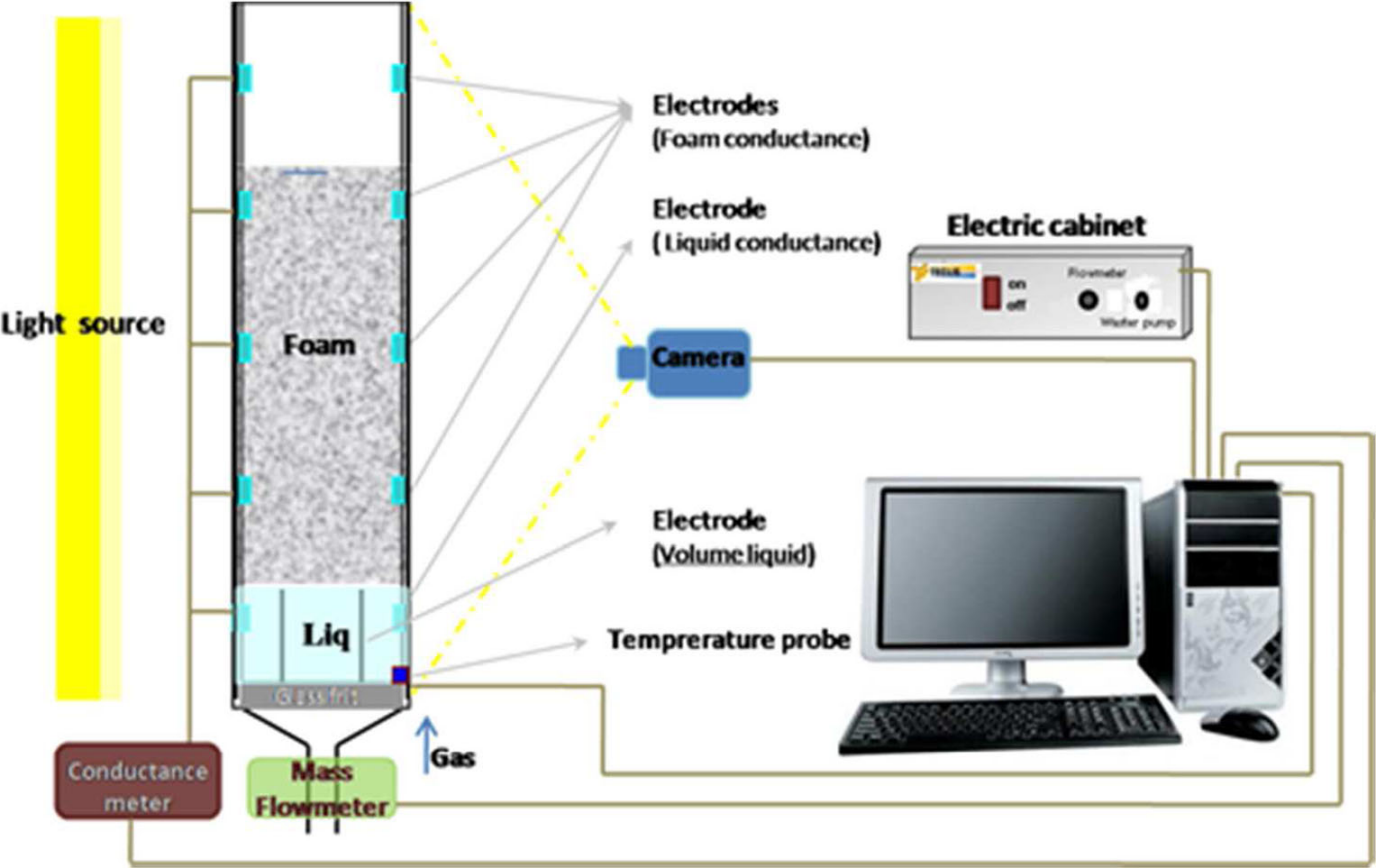
Schematic representation Foam Scan set-up showing the mass flowmeter to control the ail flow and the optical camera for monitoring the height of foam column to determine the foam volume. Liquid volume in the foam structure was obtained by the conductivity measurement along the glass column, Teclis Instrument﻿

The foaming properties of the chemicals used in the micro-emulsion phase behaviour study were tested by using the Foam Scan apparatus (IT Concept, France). Foam was generated in the apparatus by sparging nitrogen gas through a porous glass plate into a certain volume of surfactant solution (50 ± 1 cm^3^) and at a fixed gas flow rate of 16 ± 1 cm^3^/min. The gas flow stopped automatically when foam volume reached a pre-set value of 150 cm^3^. The foam volumes during the generation of foam and the subsequent foam drainage were monitored with real-time images of the foam column, which were recorded by a CCD camera. Several electrodes were attached to the foam column at different heights, which enabled the amount of liquid volume in the foam to be measured by conductivity measurements. A pair of electrodes at the bottom of the column was applied to record the amount of liquid which was outside of the foam structure. The Foam Scan instrument used in this study is displayed in Fig. 2 along with a snapshot of a foam column.

The following parameters were measured in experiments with the Foam Scan: the foam volume and the liquid volume content of foam versus time during gas sparging (foamability) and the decay of the foam volume and liquid volume content of foam after stopping gas sparging (foam stability). Foam stability was assessed by measuring the half-decay time *t*_1/2_, i.e. the time required to collapse foam volume to one half of the initial height of the foam column. Foam Scan apparatus can determine simultaneously the liquid volume in the foam structure, the measurement of other parameters such as foaming capacity (*FC*) and foam maximum density (*MD*). These data are used to analyse foam stability. The foamability of the surfactant solutions was described by the *FC* and *MD* coefficients.

The *FC* coefficient is the ratio of foam volume at the end of gas sparging to the total gas volume injected. The *FC* coefficient is higher than the unity for stable foam. When part of the injected gas does not reside in foam, the *FC* value of that experiment will be smaller than one which could be an indication of un-stability during the foam process. The *MD* coefficient was defined as a ratio of the liquid volume in the foam to the final foam volume. The maximum density provides insight in the liquid hold-up of the generated foam: the more wet the foam is, the higher the *MD* value will be [33].

### Experimental methodology

#### Salinity scan

Micro-emulsion phase behaviour tests included the salinity scan, where the phase changes from type I (oil in excess water phase) to type III (a bi-continuous oil/water phase) and then to type II (water in excess oleic phase) as the salinity increases. First, chemical systems containing surfactant, electrolyte and model oil either with or without alkali and organic acid were tested in the micro-emulsion phase behaviour experiment to identify the composition of the chemical slug and the drive for ASF flooding. In order to identify the micro-emulsion phase boundary, Winsor phase behaviour of brine-surfactant-oil systems were performed under the specific conditions of salinity, surfactant concentration and oil type, as demonstrated in Table 3.

**Table 3:** Overview of the all surfactant phase behaviour experiments by the salinity scan method

System	Surfactant conc. (wt%)	Electrolyte type	Oil type
1	0.5	NaCl	*n*-Hexadecane
2	0.5	Na_2_CO_3_/NaCl	*n*-Hexadecane
3	0.5	Na_2_CO_3_/NaCl	*n*-Hexadecane + naphthenic acid
4	1.0	NaCl	*n*-Hexadecane
5	1.0	Na_2_CO_3_/NaCl	*n*-Hexadecane
6	1.0	Na_2_CO_3_/NaCl	*n*-Hexadecane + naphthenic acid

Two types of micro-emulsion were generated: one is from a surfactant, alkaline/surfactant (AS) solution with *n*-hexadecane, and the other type is an alkaline/surfactant (AS) solution with an organic acidic mixture of *n*-hexadecane. For the salinity scan test in the phase behaviour study, the water/oil ratio was equal to one. The characteristic transition of micro-emulsion from type I to type III to type II by increasing the salinity was represented by a volume fraction diagram, which indicates the sensitivity of the surfactant solution behaviour to additional electrolytes [31]. Information relevant to the observed type of various phase behaviours (such as Winsor I, Winsor II or Winsor III) was visually observed at equilibrium conditions.

#### Foamability and foam stability

The foamability and foam stability of the different systems considered in the micro-emulsion phase behaviour study were examined by investigating the effect of several parameters. First, the effect of surfactant concentration on the stability of IOS foam in the absence and presence of the oil was investigated, where oil saturation was 5.0% by volume. Then, in order to understand the impact of oil saturation on foam stability, the generated foam containing 1.0 wt% IOS was exposed to the different volume concentrations of *n*-hexadecane. The amounts of liquid volume until the maximum value was reached were measured; these were obtained at different times depending on the surfactant concentration and oil saturation. In the next step, to demonstrate the effect of salt and alkalinity on the foam stability, the decay time of the foam column was halved, using the 1.0 wt% IOS surfactant throughout the range of salt and alkaline concentration. Finally, the effects of in situ soap generation and IFT on the stability of foam were investigated. All of the foamability and foam stability experiments performed in this study are listed in Table 4.

**Table 4:** Overview of all the foamability and foam stability experiments by the foam scan method

Experiment	Changing parameters	Surfactant conc. (wt%)	Electrolyte type	Oil type
1	Surfactant concentration	0.1 up to 2.0	NaCl	Without oil
2	Surfactant concentration	0.1 up to 2.0	NaCl	*n*-Hexadecane
3	Oil saturation	1.0	NaCl	*n*-Hexadecane
4	NaCl concentration	1.0	NaCl	Without oil
5	Na_2_CO_3_ concentration	1.0	Na_2_CO_3_	Without oil
6	Na_2_CO_3_ concentration	1.0	NaCl/ Na_2_CO_3_	*n*-Hexadecane with naphthenic acid

## Results and discussion

### Surfactant phase behaviour investigation

**Fig. 3:**
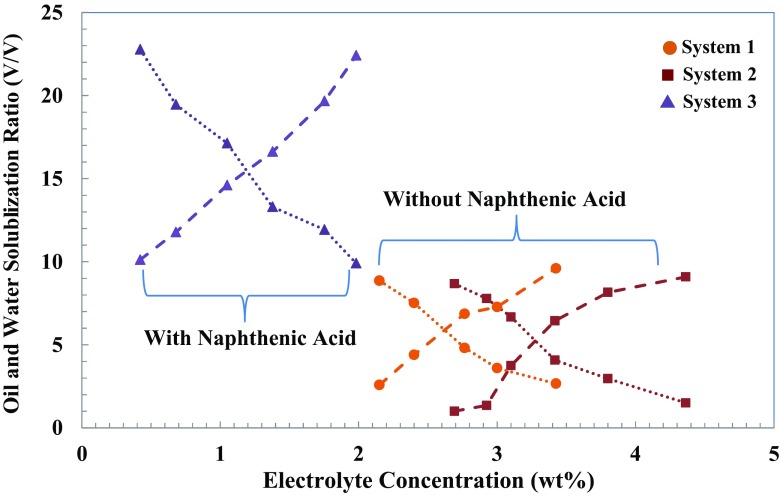
Solubilization ratio of oil and water for systems containing 0.5 wt% IOS surfactant solution contacting hexadecane model oil with a variation of NaCl concentration. *Dotted line*: connecting water phase solubilization ratio; *dashed line*: connecting oil phase solubilization ratio

**Fig. 4:**
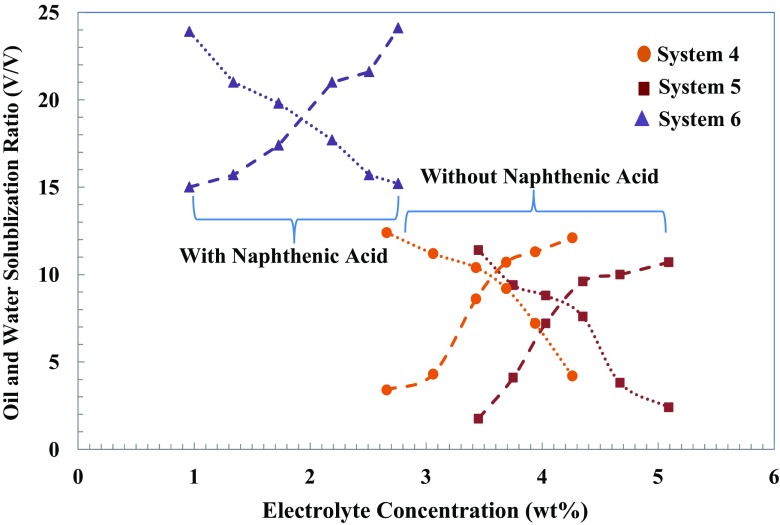
Solubilization ratio of oil and water for systems containing 1.0 wt% IOS surfactant solution contacting hexadecane model oil with a variation of NaCl concentration. *Dotted line*: connecting water phase solubilization ratio; *dashed line*: connecting oil phase solubilization ratio

Figures 3 and 4 show solubilization parameters (*V*_*o*_/*V*_*s*_) and (*V*_*w*_/*V*_*s*_) for the systems earlier presented in Table 3. The oil, brine and surfactant solubilization volumes, *V*_*o*_, *V*_*w*_ and *V*_*s*_, in the micro-emulsion phase, were estimated from the phase volumes. The figures present the solubilization parameter on the salinity of two systems containing 0.5 and 1.0 active weight percentage of IOS surfactant equilibrate with the model oil. In these two plots, the data points of the oil solubilization ratio are connected with the dashed line while the water solubilization ratio is shown by the dotted line. The intersection of the plots of *V*_*o*_/*V*_*s*_ and *V*_*w*_/*V*_*s*_ as a function of salinity gives the optimum salinity and the optimum solubilization ratio. Optimum salinity corresponds to the salinity that equal volumes of water and oil are solubilized in the middle phase in Winsor type III of micro-emulsion phase behaviour.

**Fig. 5:**
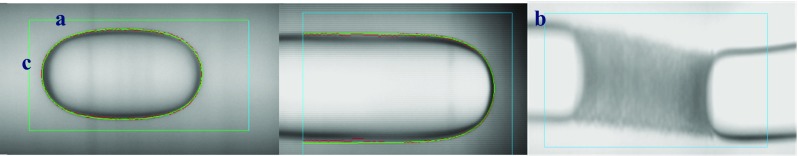
Oil droplet shapes in the range of IFT values with the aqueous solution of IOS surfactant with salinity and alkali in the capillary tube of a spinning drop tensiometer: *a* relatively low IFT (1 to 5 mN/m), *b* low IFT (1 to 10^−2^ mN/m), and *c* ultra-low IFT (less than 10^−2^ mN/m)

Optimum salinities, where the two solubilization parameters have equal values (*V*/*V*_*s*_) according to Figs. 3 and 4, solubilization parameters (measured at optimum salinities) and optimal IFT for all the examined systems are summarized in Table 5. IFTs at optimum salinity were obtained by a spinning drop tensiometer through the drop shape analysis; Fig. 5 shows the example of images used for drop shape analysis for the range of IFT values.

**Table 5:** Experimental data of the surfactant phase behaviour study for three types of chemical systems containing 0.5 and 1.0 wt% IOS surfactant at optimal conditions

Systems	Electrolyte	Oil phase	Optimum salinity (wt% NaCl)	Optimum solubilization ratio	IFT at optimum salinity (mN/m)
1	NaCl	Hexadecane	3.2	5.90	9.19E−2
2	NaCl–Na_2_CO_3_	Hexadecane	2.6	7.10	6.35E−2
3	NaCl–Na_2_CO_3_	Acidic Hexadecane	1.1	15.95	1.19E−3
4	NaCl	Hexadecane	4.1	9.75	3.37E−2
5	NaCl–Na_2_CO_3_	Hexadecane	3.5	10.50	2.9E−2
6	NaCl–Na_2_CO_3_	Acidic Hexadecane	1.9	19.90	6.86E−4

Table 5 shows the phase behaviour results comparing the optimum salinity, solubilization ratio and IFT values of systems with and without alkali contacting with and without acidic model oil. The measured data indicate that only the addition of both alkalis and surfactant to the water phase does not reduce the IFT substantially; however, a much greater IFT reduction can be obtained by the generation of in situ soap. As shown in Fig. 4, solubilization ratio values (*V*/*V*_*s*_) exceeding 10 were obtained for all systems containing 1.0 wt% surfactant. When the optimum solubilization ratio is equal to or larger than 10, then IFT at optimum salinity is in the order of 10^−3^ mN/m or less [43]. This IFT reduction is sufficiently low to mobilize the trapped residual oil by capillary forces. However, for aqueous solutions containing 0.5 wt% of IOS surfactant, in Fig. 3, we can only see solubilization ratio higher than 10 where in situ soap generation-assisting IFT reduction exists in a system containing naphthenic acid. As we made a goal of designing a chemical formulation for ASF flooding, this data indicates the impact of the presence of alkalinity, soap generation and surfactant concentration on a range of optimum salinity, solubilization parameters and IFT values.

### Bulk foam stability

#### Effect of surfactant concentration and oil saturation

**Fig. 6:**
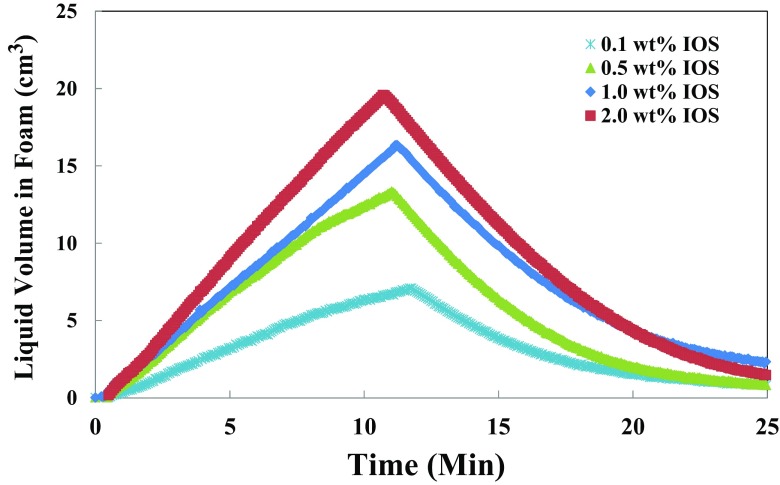
Effect of IOS surfactant concentration in the absence of an oil phase illustrated by a change in the liquid volume of foam during foam generation and after termination of gas sparging as a function of time. The initial liquid volume of the generated foam is 50 cm^3^ and the maximum volume of generated foam are illustrated

**Fig. 7:**
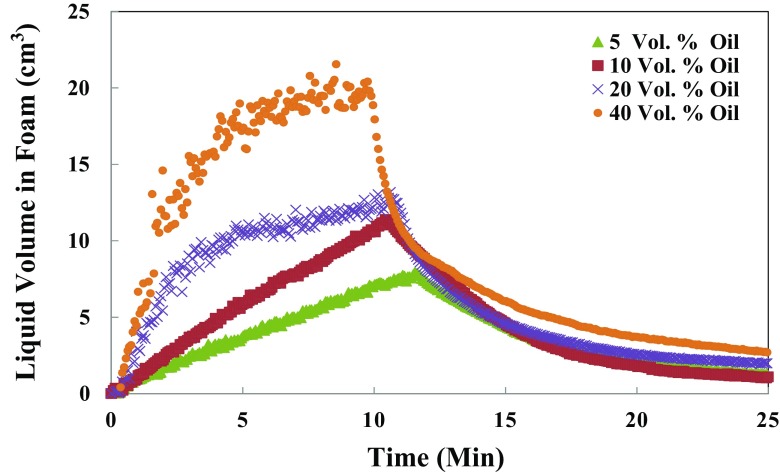
The change in the liquid volume of foam during foam generation and after termination of gas sparging as a function of time for the various oil saturations (volume percent). The initial liquid volume of the generated foam is 50 cm^3^, and the maximum volume of generated foam from 1.0 wt% IOS surfactant in the presence of the oil phase using the foam scan is illustrated

In this section, we investigate the effect of the surfactant concentration with and without the contacting oil as well as the effect of oil saturation on foamability and foam stability. Firstly, to investigate the effect of the IOS surfactant concentration on the stability of foam, the concentration was varied from 0.1 to 2.0 wt%, but in all the other experiments, the IOS concentration was kept constant at 1.0 wt%. Foam drainage, i.e. the decay of liquid volume in the foam as a function of time, is depicted in Figs. 6 and 7 in the absence and presence of an oleic phase, respectively. Figure 6 shows the evolution of liquid volume hold-up in the foam structure for the different surfactant concentrations as a function of time during foam generation and drainage after switching off the air sparging.

**Fig. 8:**
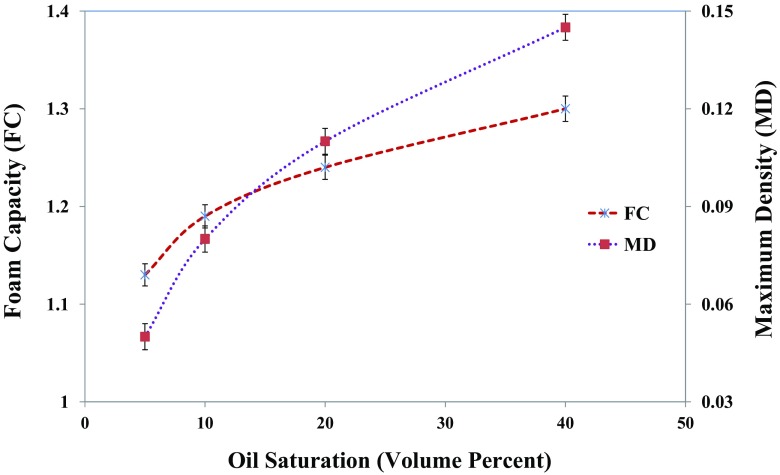
Effect of the different oil saturations on the foam maximum density (*MD*) and foam capacity (*FC*) of 1.0 wt% IOS generated foam

Data in Fig. 6 show that IOS foam grows linearly with time during foam generation. The straight line in the foam liquid–volume profile indicates a stable build-up of foam volume, and thus an IOS foam evolution is not affected by the destruction processes, such as coalescence and Ostwald ripening during foam generation [4]. Figure 6 also shows that liquid hold-up increases with surfactant concentrations, which can be explained by the fact that with increasing surfactant concentration the bubble size decreases which results in the intense fine foam texture, as visually observed during the experiments. Within longer periods of foam stability, it was observed that the average bubble size increases with decreasing surfactant concentration due to bubble coalescence. Though the maximum amount of liquid (*V*_*L*,*max*_) in the foam for the higher surfactant is larger, the time taken to reach the *V*_*L*,*max*_ is correspondingly shorter. This implies a larger foamability for the higher surfactant concentration is due to the higher amount of adsorbed surfactant and the larger transport rate of surfactant to the aqueous phase/gas phase interface. This leads to the strength of electrostatic double-layer effect and also Gibbs–Marangoni effect, which both results in a more stable foam at the higher surfactant concentration [21]. Figure 7 shows similar experiments, in the presence of oil with various levels of oil saturation in the foam column of 1.0 wt% IOS surfactant. To gain further insight into the effect of oil saturation on foam properties, the foam capacity (*FC*) and the maximum density (*MD*) were measured as demonstrated in Fig. 8.

**Fig. 9:**
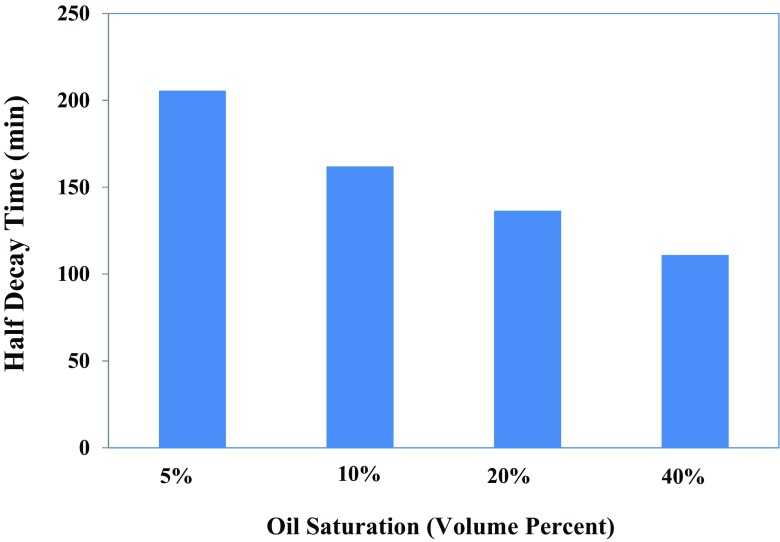
Effect of different levels of oil saturations of *n*-hexadecane on the foam half-decay time generated by 1.0 wt% IOS

For the experiment in the presence of oil, the amount of liquid entrained inside the foam structure raised as the oil saturation added (Fig. 7). During the foam generation, part of oil enters into foam lamellae and thickens the plateau borders leading to the transport of oil within foam. This observation can be supported by the variation of *FC* and *MD* as shown in Fig. 8. A higher liquid volume in the foam is expected to lead to a lower drainage rate for the same surfactant solution in similar experimental conditions. However, as indicated in Fig. 9 that depicts half-decay time of 1.0 wt% IOS surfactant solution contacting with the range of oil saturation, the foam generated in the presence of a higher oil saturation has a lower half-decay time. Thus, the higher liquid volume in the foam structure in turn led to a larger drainage rate and a faster decline of foam volume compared to the generated foam interacting with the lower oil saturation. This could be due to the penetration of portion of the oil present in the foam lamellae and plateau borders to the gas–surfactant interface, which leads to the rupture of the foam films. This mechanism may explain the fact that the destabilizing effect of oil increases with the increase of oil saturation under the static foam condition.

**Fig. 10:**
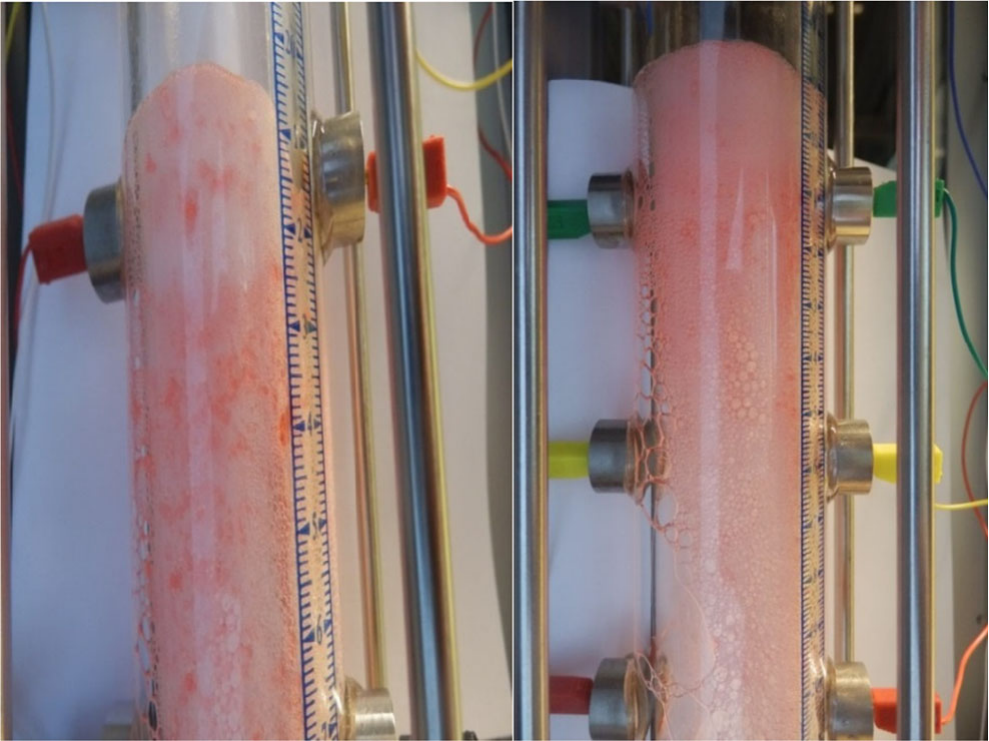
Foam column stabilized by IOS surfactant in the presence of *n*-hexadecane. The oil phase was coloured red to visualize. The left-hand image was taken at an early time in the foam decay and the right-hand one was taken at a later time. The images confirm the capability of the generated foam to be tolerant to the oleic phase while drained liquid stayed in the column

**Fig. 11:**
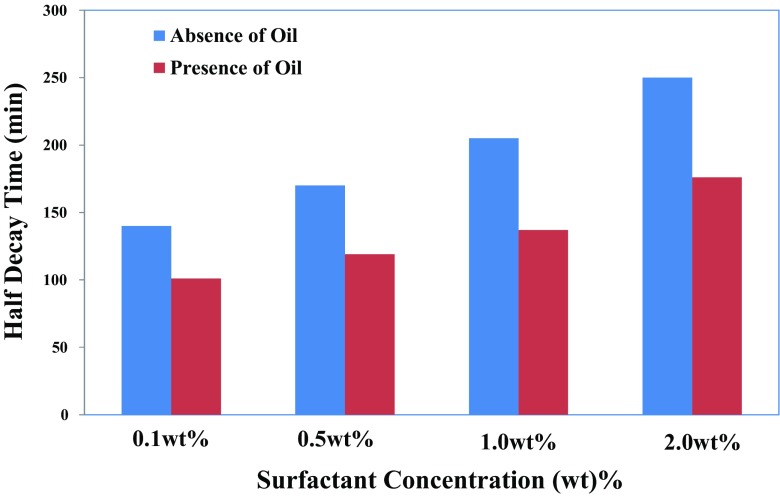
Effect of IOS surfactant concentration on the foam half-decay time in the absence and presence of 5.0 vol% of *n*-hexadecane

Figure 10 displays visually the foam columns generated by 1.0 wt% IOS in the presence of normal hexadecane. The oleic phase was coloured red for the visualization. The image on the left was taken at an early point in the foam decay and the image on the right-hand side was taken at a later point. As can be seen, the created foam can carry large portion of the oil upward, which results in a relatively uniform distribution of oil in the body of foam. The decay of IOS foam was continued by coalescence of bubbles at the middle of the column causing a local change in the foam texture. The snapshot of the foam column clearly shows that, although foam texture in the latter point of the experiment is coarse, the foam is still stable by holding the oil in the body of foam. Thus, for the IOS foam (in the right-hand image) after gas sparging was terminated, the foam column remained stable for a relatively long time as can also be inferred by the *t*_1/2_ in Fig. 11.

**Fig. 12:**
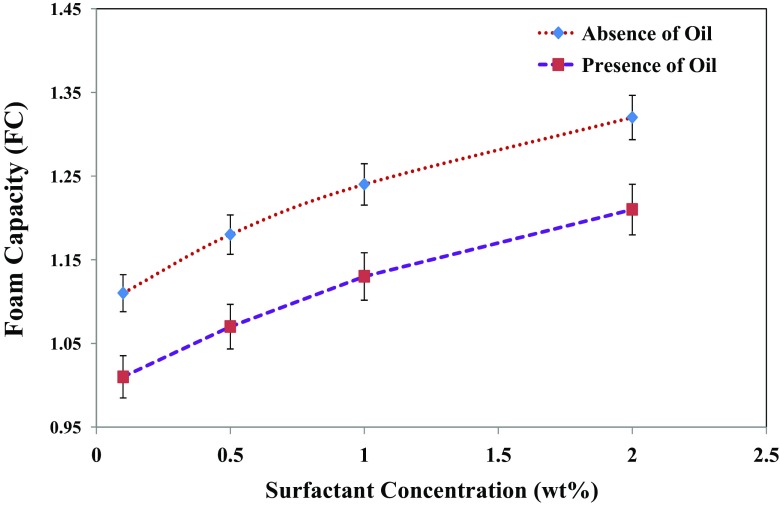
Effect of the IOS surfactant concentration on the foaming capacity (*FC*) in the absence and presence of 5 vol% *n*-hexadecane. The initial surfactant solution volume of the generated foam is 50 cm^3^ and in the case of the presence of *n*-hexadecane, oil saturation is 5 vol%

Figure 11 shows that half-decay time, *t*_1/2_, in the presence of oil is systematically lower than in the absence of oil, and it increases with surfactant concentration. The data in Fig. 12 also show the *MD* coefficient of freshly generated foams as function of surfactant concentration in the absence and presence of *n*-hexadecane. As shown, the *FC* coefficient is larger than unity, even for foam stabilized by a low surfactant concentration of 0.1 wt% (Fig. 12). Hence, this coefficient for foam in the presence of oil is systematically lower than that in the absence of oil. The difference in the *FC* coefficients can be attributed to the gas sparging time. Recall that *FC* coefficient was defined as a foam volume at the end of gas sparging divided by the total volume of gas injected. This infers that injection of a larger volume of gas leads to a smaller value of the *FC* coefficient [33]. Therefore, both coefficients increased with surfactant concentration and oil saturation.

#### Effect of salinity and alkalinity

**Fig. 13:**
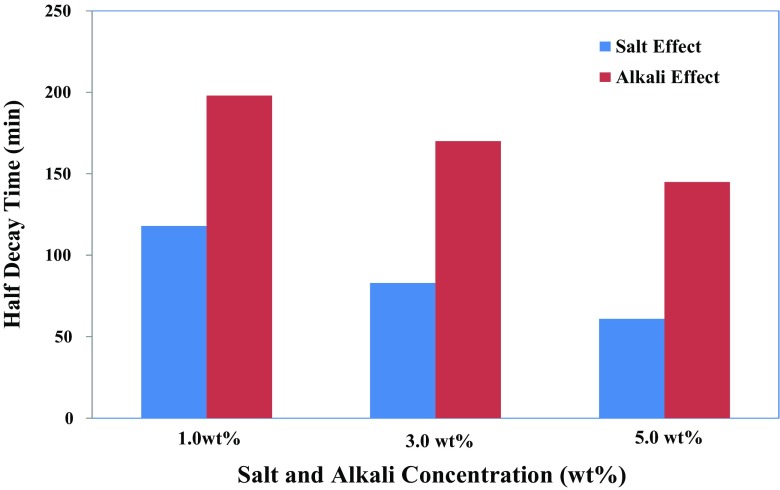
Effect of salt (NaCl) and alkali (Na_2_CO_3_) concentration on the foam half-decay time of 1.0 wt% IOS surfactant

**Fig. 14:**
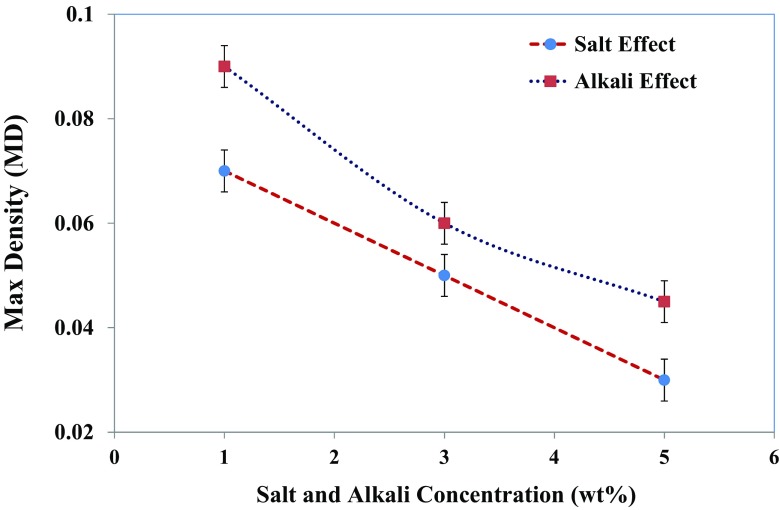
Effect of salt (NaCl) and alkali (Na_2_CO_3_) concentration on the maximum density (MD) of generated foam with 1.0 wt% IOS surfactant

Illustrated in Fig. 13 is the effect of salinity and alkalinity on the foam stability in the absence of oleic phase. Concentrations of salt/alkaline increase up to 5.0 wt%, which is the range of electrolyte concentration obtained from the micro-emulsion phase behaviour study (see the “Surfactant phase behaviour investigation” section). From this figure, it can be seen that the addition of salt (NaCl) and alkali (Na_2_CO_3_) to the IOS foaming system can have an effect on the reduction of foamability and foam stability. Figure 14 shows that the *MD* of the generated foam decreases; such effects have been associated with the cationic–anionic-type interaction between the anionic moiety of the IOS surfactant and cation ion of the salt and alkali. This type of interaction causes the screening of the repulsive forces between the ionic head groups and reducing the surface potential on the gas–liquid interfaces. Consequently, this causes a reduction in the repulsion between the surfactant layers, between the opposing film interfaces, and thus decreasing double-layer repulsion which in turn favours film drainage.

#### Effect of in situ soap generation

**Fig. 15:**
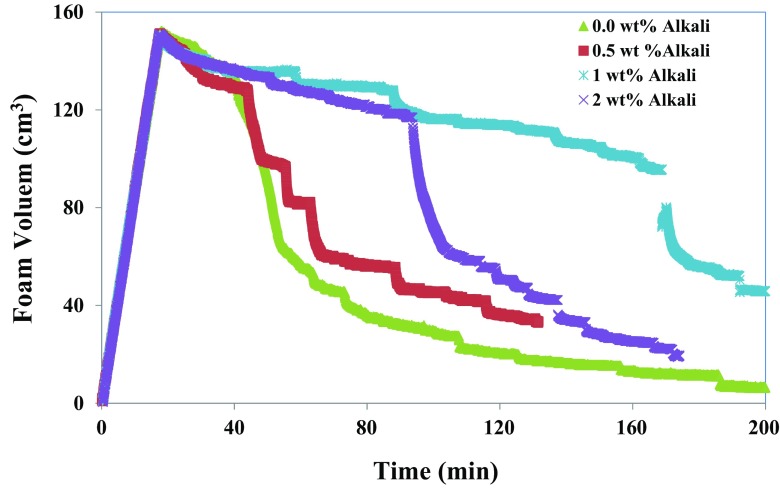
The effect of in situ soap generation on the stability of foam is illustrated. Foam volume as a function of time for 0.5 wt% IOS foam contacting *n*-hexadecane in the absence and presence of naphthenic acid (decanoic acid)

Surfactant solution containing 0.5 wt% IOS and 1.0 wt% NaCl with different concentrations of Na_2_CO_3_ were used to study the effect of in situ soap generation on foam drainage. Figure 15 shows the foam volumes versus time for the different alkali concentrations in the aqueous phase contacting with *n*-hexadecane containing decanoic acid. Increasing the alkali concentration from 0.5 to 1.0 wt% resulted in an enhanced foam stability. This can be explained by the fact that higher alkalinity means more natural surfactant in the system due to in situ soap generation. However, for the surfactant solution containing 2.0 wt% alkali, the drainage rate is larger and the extent of stability is smaller than for a system containing 1.0 wt% alkali. This suggests that the effect of the alkali is reversed due to a large amount of in situ soap generation. This could be due to the fact that the liquid–gas interface is more mobile at a lower surface tension (higher in situ soap), which tends to increase the rate of liquid drained out of the plateau border. This reduction in liquid occurs during the initial liquid holdup as well as during drainage. At lower surface tensions, the capillary suction at the plateau border (which is against gravity) is smaller and, thus, the rate of foam drainage is greater. Therefore, uneven thinning and instabilities of the foam film might happen, which will cause acceleration of the film drainage and rupture.

The reason for the observed behaviour is not completely clear. This observation could be also interpreted by the rapid spreading of oil droplets that have a low surface tension over the lamella. The spreading oil by augmenting the curvature radius of the bubbles lowers the surface elasticity and surface viscosity [21]. This can subsequently cause a rupture in the foam structure by creating weak spots. Therefore, the interfacial film loses its foam-stabilizing capability and foam destruction occurs at a significantly low surface tension.

#### Interpretation by phenomenological theories

Table 6 displays the entering, spreading and bridging the coefficients and the lamella number obtained by combining the measured ST and IFT between surfactant solution/air, model oil/air and surfactant solution/model oil. The purpose of obtaining these phenomenological parameters was to gain insight into any correlation between the classical theory and the bulk foam stability in the presence of oil. All the surfactant solutions exhibited a positive entering coefficient (*E* > 0), indicating favourable conditions for *n*-hexadecane and acidic *n*-hexadecane to enter the gas–water interface. Thus, foam stability in the presence of oil will be determined by the magnitude and sign of the spreading *S* and bridging *B* coefficients (see also Table 1). Among the systems studied, systems 1 and 2 provide the negative spreading coefficients, but these systems showed the largest positive *B* coefficients. This indicates that the generated foam should be relatively stable in the presence of *n*-hexadecane, in a good agreement with the observed decay behaviour in Fig. 11.

**Table 6:** Entering, spreading and bridging coefficients and lamella number for different studied systems in presence of *n*-hexadecane

System	Composition	Entering coefficient (mN/m)	Spreading coefficient (mN/m)	Bridging coefficient (mN/m)^2^	Lamella number
1	IOS/hexadecane	22.85	−2.59	784.89	0.422
2	IOS-alkali/hexadecane	30.64	−6.48	1106.71	0.304
3	IOS/acidic hexadecane	13.62	13.62	440.59	0.812
4	IOS-alkali/acidic hexadecane	10.74	10.74	468.23	4.172

Surfactant concentration was fixed at 1.0 wt%

Foam stability can be further examined by comparing the value of the lamella number. Systems 1 and 2 exhibit a lamella number of smaller than one, which corresponds to type *A* foam. We recall that a type *A* foam is stable in presence of oil with a negative *S* coefficient (see Table 2). However, this is not in line with the calculated *E* and *S* coefficients in Table 6. It is also not consistent with the observed foam stability in Fig. 11, particularly for the case of pure *n*-hexadecane (without naphthenic acid), which were found to be rather sensitive to the oleic phase. The spreading coefficients calculated for the acidic model oil were positive for systems 3 and 4 regardless of the presence of alkali and in situ soap generation. In theory, in such a situation the oil could spread over the gas-liquid surface and break the foam film, however, according to measured half-decay time, the generated foam was fairly stable (see Fig. 11). We recall that if the spreading coefficient was negative, oil would remain as droplets at the interfacial surfaces and thus attains a necessary condition to stabilize foam.

Systems 3 and 4, in the presence of acidic oil, exhibited positive entering and spreading coefficients which indicate type *C* foams. However, for these two systems, foam stability does not seem to be governed by this type of classification. On the other hand, visual inspection of the foam-column experiments indicated that foam made using surfactant formulations can emulsify the acidic model oil into plateau borders of the foam structure. Thus, system 4 exhibited type *B* foam behaviour, which indicates that foam stability in the presence of soap generation could be attributed to transport properties of oil droplets within the foam. Type *B* foams have the capacity to carry more oil than type *A* or type *C* foams by transporting emulsified oil droplets inside the foam structure [39].

For all IOS foams generated in the presence of oil, the bridging coefficient was high and positive, which implies that the bridging mechanism can trigger a film rupture. Lower magnitude of the entering and bridging coefficients for system 4 than system 3 as presented in Table 6 indicates that IOS foam can generate more stable foams when mixed with soap generated by the interaction of alkali and naphthenic acid present in the oleic phase. Thus, we could bring to a close that a negative spreading coefficient is not a necessary condition for stable foam, and the stability of foam in the presence of oil could be attributed to interfacial properties and oil transport characteristics of the foam plateau borders and the foam lamellae.

## Conclusions

- An extensive laboratory study of the micro-emulsion phase behaviour, interfacial properties and foam stability characterization was presented to evaluate the properties of chemical slug/drive for the ASF flooding EOR. A surfactant formulation, giving ultra-low IFT at the optimum salinity and with fairly good foaming characteristics, was experimentally achieved.
- The micro-emulsion phase behaviour study of a particular system in this work demonstrated the impact of the presence of alkalinity, in situ soap generation and surfactant concentration on a range of optimum salinity, oil/water solubilization parameters and IFT values. A water and oil solubilization ratio of 10, as a criterion to get sufficiently low IFT for a high tertiary oil recovery, was met by all the systems containing 1.0 wt% surfactant. However, for the system of 0.5 wt% of surfactant, this criterion was only met for the system interacting with acidic oil, where there is in situ soap generation assisting IFT reduction.
- Foam drainage with and without the presence on oleic phase was influenced by the physico-chemical properties of surfactant solutions and the tolerance of the generated foams to capillary suction pressure and bubble coalescence. Our results showed that although the amount of liquid entrained inside the foam structure raised as the oil saturation added, the presence of higher oil saturation increases coarsening rate of foams.
- The effect of alkalinity on lowering foam stability could be attributed either to screening the repulsive forces between the ionic head group resulting from cationic–anionic-type interaction and decreasing double-layer repulsion or to the change in the micelle structure from spherical micelles to other more complex structures.
- A large amount of in situ soap generation resulted in diminishing foam stability. This observation could be interpreted by the rapid spreading of oil droplets that have a low surface tension over the lamella. The spreading oil, by augmenting the curvature radius of the bubbles, decreases the surface elasticity and surface viscosity. This subsequently can cause a rupture in the foam structure by creating weak spots over the interfacial lamella film.
- Less foam stability at significantly low IFT between the aqueous phase and oleic phase can also be explained by the fact that the gas–liquid interface is more mobile at a lower surface tension, which tends to increase the rate of liquid drained out of the plateau border. At lower surface tensions, the capillary suction at the plateau border (which is against gravity) is smaller and, therefore, the rate of foam drainage is greater. Thus, uneven thinning and instabilities of the film might happen, which will cause acceleration of film drainage and lamellae rupture.
- The classical phenomenological parameters, such as spreading and entering coefficients, have been used with some success and similarities in trend; however, foam performance by these parameters did not correlate for the foam stability to oil for most of the experiments.
